# Determination of Phytochemical Contents in Extracts from Different Growth Stages of *Oroxylum indicum* Fruits Using HPLC-DAD and QAMS Methods

**DOI:** 10.3390/molecules28196837

**Published:** 2023-09-28

**Authors:** Piyanuch Rojsanga, Stefan Schwaiger, Hermann Stuppner, Pongtip Sithisarn

**Affiliations:** 1 Department of Pharmaceutical Chemistry, Faculty of Pharmacy, Mahidol University, Bangkok 10400, Thailand; piyanuch.roj@mahidol.ac.th; 2 Institute of Pharmacy/Pharmacognosy, CCB—Center for Chemistry and Biomedicine, University of Innsbruck, 6020 Innsbruck, Austria; hermann.stuppner@uibk.ac.at; 3 Department of Pharmacognosy, Faculty of Pharmacy, Mahidol University, Bangkok 10400, Thailand

**Keywords:** *Oroxylum indicum*, HPLC, QAMS, flavone

## Abstract

Flavones are major compounds found in several parts of *Oroxylum indicum* (*O. indicum*). The quantification of multiple components by one marker (QAMS) method and the high-performance liquid chromatography (HPLC) method were developed for the quantitative analysis of extracts from the young fruits, green mature fruits, dry pod coats and seeds of *O. indicum*. Oroxin A, oroxin B and chrysin-7-*O*-glucuronide were identified in the *O. indicum* extracts. Oroxylin A and 5-hydroxymethylfurfural were isolated and structurally identified from the pod coat and young fruit extracts, respectively. From the HPLC analysis of the seven major flavones in the extracts, baicalin was the major compound in all extracts investigated (0.4–11% *w*/*w* of the extract). All flavone contents were low in the young fruit extract (<1% *w*/*w* of the extract). The green mature fruit and dry pod coat extracts showed similar constituent compositions. They contained small amounts of baicalin and oroxylin A, which were found only in these two extracts. Oroxylin A could be used as a marker to indicate the maturity of *O. indicum* fruits, while 5-hydroxymethylfurfural could be used as a marker for the young fruits. Baicalin was found to be a suitable single marker to calculate the contents of all flavones in the *O. indicum* extracts.

## 1. Introduction

*Oroxylum indicum* (L.) Benth. ex Kurz. or pheka is a member of the Bignoniaceae family. The young fruits are the edible stage of this plant, and are commonly consumed as vegetables. The seeds are the well-known as the compositions of a herbal drink for the treatment of aphthous mouth ulcers and thirst. Various parts of this species are used for medicinal purposes. The seeds are used as a laxative and for the treatment of tonsil pain [1], the treatment of epilepsy [2], as an analgesic or antitussive and for the treatment of bronchitis, pharyngitis, or other respiratory problems [3]. Additionally, the seeds are used for the treatments of gastritis ulcers and tumors [4], while the mature fruits exhibit anti-helminthic and stomachic effects [5], show beneficial effects against bronchitis and cardiac ailments [6,7] and possess an antidiarrheal effect [8]. The root and the root bark have been used for hepatoprotective purposes and also for anti-jaundice, antidiarrheal and antidiabetic effects [4,9,10]. Our previous study showed that extracts from the fruits and the seeds of *O. indicum* possess in vitro antioxidant effects determined via a DPPH assay, as well as in vitro antibacterial effects against zoonotic *Staphylococcus intermedius* and *Streptococcus suis* [11]. The in vitro DPPH scavenging effects, the total phenolic and total flavonoid contents and an LC-MS chromatogram of different plant parts including flowers, pedicels, leaves, stalks, seeds and fruits, tissue-cultured plants and callus cultures of *O. indicum* were also reported [12]. The fruit extract at a concentration of 5 mg/mL caused morphological changes in bacteria by disrupting the bacterial cell walls, inducing the leakage of the cellular content and generating an abnormal accumulation of cells, suggesting that its underlying mode of action is the disruption of the cell membrane and abnormal cell aggregations [13]. Flavonoids were found to be major compounds in *O. indicum*. The stem barks have been reported to mainly contain baicalein, chrysin and oroxylin A [14,15], while the root barks of this plant were reported to contain oroxylin A and baicalein as major compounds [16,17]. Baicalein was found to be a major compound in the fruits [18], while baicalein, chrysin, oroxin B, oroxin A and chrysin-7-*O*-diglucoside have been reported from the seeds [19]. The major components in the leaves were found to be baicalein, scutellarein and their glucuronide compounds [20]. From our previous work, besides three major compounds, baicalin, baicalein and chrysin, which were quantitative analyzed using the developed HPLC method [13], there were another three peaks that could be found in the extracts from *O. indicum* such as the seeds and fruits. These three unidentified peaks, along with any other unknown peak in *O. indicum* extracts, should be identified and quantitatively analyzed. The quantification of multiple components by one marker (QAMS) has been reported as a method for the analysis of multiple components in an extract using only one single marker compound, such as the analysis of ginsenosides in *Panax ginseng* and *P. notoginseng* via the HPLC method using ginsenoside Rb1 as a single marker [21]. There are seven major compounds in *O. indicum* extracts which consume time, cost and labor. Therefore, the aims of this study were to identify and quantitatively analyze the major flavones in the extracts from several plant parts of *O. indicum* including the young fruits, green mature fruits, dry pod coats and seeds. The quantification of multiple components by one marker was also developed to find a single marker that could be used for evaluating the amounts of all flavones in the *O. indicum* extracts.

## 2. Results and Discussion

### 2.1. Identification and Isolation of Flavones

#### 2.1.1. Identification of Flavones

**Figure 1:**
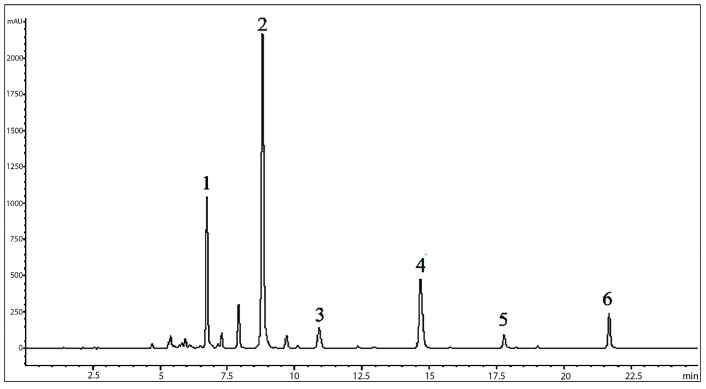
HPLC chromatogram of the analysis of *O. indicum* seed extract from Lampang province; peak 1 = oroxin B (**1**), peak 2 = oroxin A (**3**), peak 3 = baicalin (**2**), peak 4 = chrysin-7-*O*-glucuronide (**4**), peak 5 = baicalein (**5**), peak 6 = chrysin (**6**) at 285 nm.

**Figure 2:**
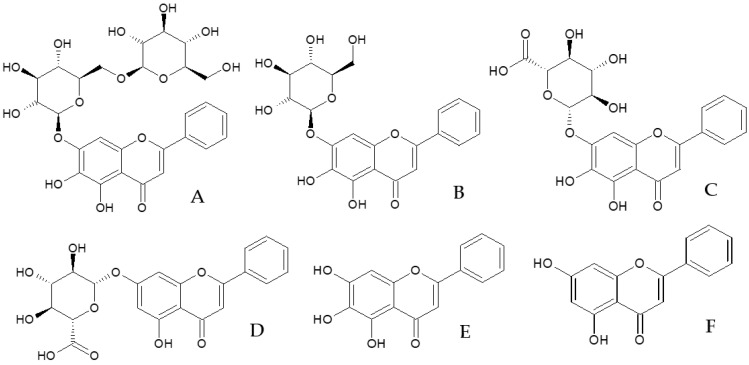
Chemical structures of major flavones in *O. indicum* seed extract; (**A**) = oroxin B (**1**), (**B**) = oroxin A (**3**), (**C**) = baicalin (**2**), (**D**) = chrysin- 7-*O*-glucuronide (**4**), (**E**) = baicalein (**5**), (**F**) = chrysin (**6**).

An LC-MS analysis of *O. indicum* extracts from various plant parts, including the seeds, the young fruits, the mature fruits and the dry pod coat, suggested the presence of six major flavones in the extract. Oroxin B (**1**), oroxin A (**3**) and chrysin-7-*O*-glucuronide (**4**) were detected in the seed extract from Lampang province with a retention time of 6.73, 8.81 and 14.67 min, respectively (Figure 1). The mass spectra of oroxin B, oroxin A and chrysin-7-*O*-glucuronide showed molecular mass ions at *m/z* [M+H]^+^/[M-H]^−^ 595.2/593.0, 433.2/431.0 and 431.2/428.8, indicating molecular weights of 594, 432 and 430, respectively. The retention times and mass spectra of these three compounds were identical with the data of the reference compounds analyzed using the same procedure. Other major compounds found in the seed extract were baicalin (**2**), baicalein (**5**) and chrysin (**6**). The chromatographic, spectroscopic and spectrometric data are shown in Table 1, and the chemical structures of all six compounds are in Figure 2.

#### 2.1.2. Additional Constituents Present in Extracts of *O. indicum*

**Figure 3:**
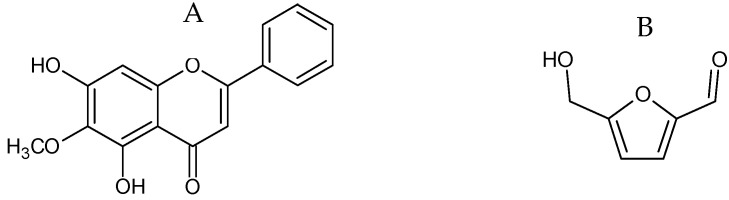
Chemical structure of compound OI1 (oroxylin A) (**7**) (**A**) and compound OI2 (5-hydroxymethylfurfural) (**8**) (**B**).

The HPLC chromatograms of the mature fruit and dry pod coat extracts showed an additional compound (OI1) with a retention time of 27.82 min. This compound was isolated using the chromatographic techniques as mentioned in Section 3.3.2.1, then structurally identified using spectroscopic and spectrometric techniques. According to the UV spectrum, the ^1^H-NMR and ^13^C-NMR data and the mass spectra, it was identified as oroxylin A (**7**) (Figure 3A). In contrast, the HPLC chromatogram of the young fruit extracts exhibited the constituent OI2, which eluted at a retention time of 14.61 min. This compound was also isolated as is mentioned in Section 3.3.2.2, then structurally identified. According to the UV spectra, the ^1^H-NMR and ^13^C-NMR data and the mass spectra, compound OI2 was identified as 5-hydroxymethylfurfural (**8**) (Figure 3B).

Oroxylin A (**7**) was previously reported in various parts of *O. indicum* including the root [16,22], root bark [23], stem bark [14,23,24,25,26], leaves [20,27] and young fruit pods [28], while oroxylin A-7-*O*-glucuronide was also found in this plant [29]. Oroxylin A was reported to inhibit xanthine oxidase with an IC_50_ of 49 µM, and with a low inhibitory effect on superoxide radical generation in HL-60 cells. However, the effects were lower than the effects from chrysin [28]. Oroxylin A was also reported to potently inhibit nitric oxide synthase (iNOS) and cyclooxygenase-2 (COX-2) in RAW 264.7 cells [30]. This compound was reported to be active against brine shrimp with LC_50_ 36 μg/mL, with antibacterial effects against both gram-positive and gram-negative bacteria, with an MIC value of 8 mg/mL [24]. Oroxylin A decreased mature adipocyte cell viability and enhanced TNF-α secretion, as well as lipolysis, and decreased Akt phosphorylation in mature adipocytes, suggesting its anti-obesity effect [31]. Oroxylin A was reported to promote anti-allergic effects by decreasing inflammatory cells and reducing the expression and secretion of Th2 cytokines [32]. It was also reported as the most active compound to inhibit NF-κB activation with IC_50_ 3.9 µM [25]. Oroxylin A inhibited the replication of SARS-CoV-2 (COVID-19) in in vitro and in vivo experiments [27,33]. This current experiment suggested the green mature fruits and dry pod coats as the sources of oroxylin A; therefore, these two plant parts, which normally are waste products and are not edible, could be the source of this compound for useful purposes in the future.

5-hydroxymethylfurfural (**8**) can be found in low amounts in various food products including honey, fruit juices, UHT milk, vinegars, jams, alcoholic products and biscuits [34]. It can be used as an indicator for the evaluation of storage time and heat damage from the sugar breakdown, which increases with temperature and storage time [34,35,36]. This compound was also used as an indicator of the degree of coffee roasting [37]. 5-Hydroxymethylfurfural has been previously reported in the roots of *O. indicum* via GC-MS analysis [34].

### 2.2. Quantitative Analysis of Flavone Contents

**Figure 4:**
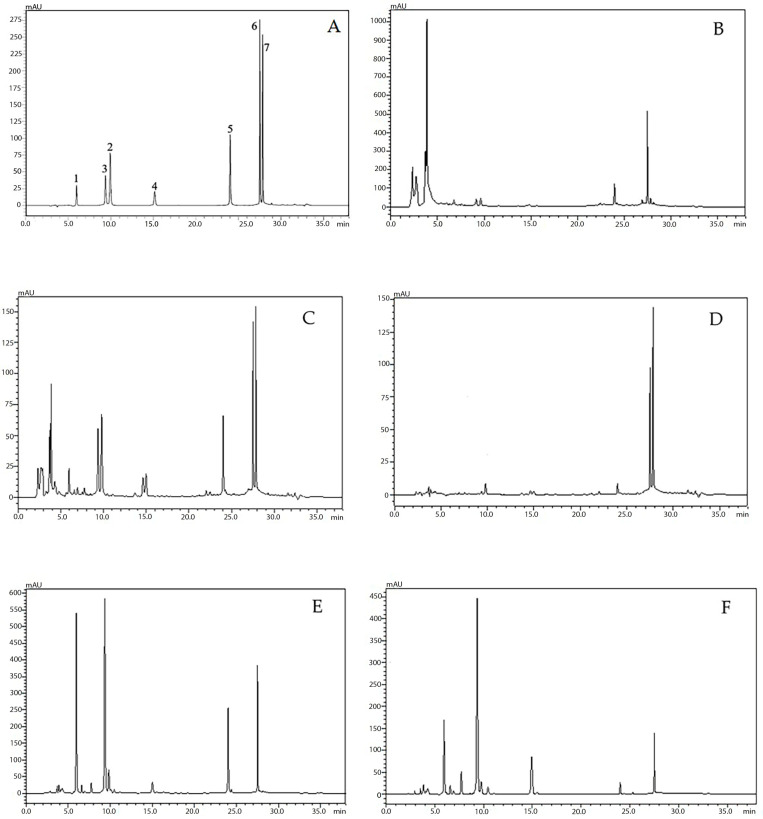
HPLC chromatogram of standard mixture used in quantitative analysis of *O. indicum*; standard mixture (**A**); peak 1 = oroxin B (**1**), peak 2 = baicalin (**2**), peak 3 = oroxin A (**3**), peak 4 = chrysin-7-*O*-glucuronide (**4**), peak 5 = baicalein (**5**), peak 6 = chrysin (**6**), peak 7 = oroxylin A (**7**), young fruit extract from Lampang (**B**), GFC = green fruit extract from Chiang Rai (**C**), dry pod coat extract from Chiang Rai (**D**), SC = seed extract from Chiang Rai (**E**). SL = seed extract from Lampang (**F**).

Using the optimized conditions, the HPLC method was validated using the specific parameter according to ICH guideline 2002, composed of linearity, accuracy and precision [38]. The peaks of seven compounds composed of oroxin B (**1**), baicalin (**2**), oroxin A (**3**), chrysin-7-*O*-glucuronide (**4**), baicalein (**5**), chrysin (**6**) and oroxylin A (**7**) were found at the retention times of 5.92 ± 0.11, 9.35 ± 0.11, 9.75 ± 0.11, 15.00 ± 0.10, 24.04 ± 0.03, 27.50 ± 0.01 and 27.85 ± 0.03 min, respectively (Figure 4).

#### 2.2.1. Method Validation

##### Linearity and Peak Purity

The linear detector responses of standard solutions of oroxin A, chrysin-7-*O*-glucuronide, baicalein, chrysin and oroxylin A were determined via the analysis of five concentration levels, while six levels of concentration were used for standard solutions of oroxin B and baicalin, each injected in triplicate. The linearity ranges of each compound are shown in Table 2. The correlation coefficient (r) values for all seven compounds were ≥0.990, confirming the linearity of the method. The peak purity was investigated using Lab Solutions software v5.6 (Shimadzu, Tokyo, Japan), revealing that the purity of each peak was ≥0.950.

##### Precision

The precision of the method was evaluated via the analysis of six independent prepared solutions of two different extracts (the seed extracts from Lampang province and the aged pod coat extract). The concentrations of six components (oroxin B, baicalin, oroxin A, chrysin-7-*O*-glucuronide, baicalein and chrysin) were calculated from the standard additions of seed extracts, while the concentration of oroxylin A was calculated from the standard addition of the pod coat extract. Then, the relative standard deviation percent (%RSD) of each compound concentration was calculated. It can be seen that the %RSD values for all analytes were at or below 1.5 (Table 2). The method can therefore be regarded as precise.

##### Accuracy

The accuracy of the method was confirmed via the determination of recovery. The recovery of active components from the extract was performed on samples spiked with three concentration levels of each compound in different ranges (Table 2). The recoveries at all three concentrations were close to the theoretical amounts (percentage of recovery was in the range of 95–105%). The method can therefore be regarded as accurate.

#### 2.2.2. Quantitative Analysis of Flavone Contents

The system suitability of the HPLC method was evaluated by analyzing three parameters including resolutions (>1.6), tailing factors (<1.4) and the %RSD of the peak area (<0.5). It was found that all parameters were acceptable.

After the system suitability was evaluated, the HPLC method was used to analyze the amounts of 7 compounds in the extracts of *O. indicum*. As shown in Table 3, only low amounts of baicalin, oroxin A, baicalein and chrysin could be detected in the young fruit extract (<1% *w*/*w* of the extract), while oroxin B, chrysin-7-*O*-glucuronide and oroxylin A could not be detected. The content of all compounds seemed to increase when the fruits grew and became mature. Baicalin was available in around 2.77% *w*/*w* of the extract, while chrysin and oroxylin A were found in around 1% *w*/*w* of the extract. However, three other compounds (oroxin B, oroxin A, chrysin-7-*O*-glucuronide) were still available in low amounts (<1% *w*/*w* of the extract). The seeds from both provinces, Lampang and Chiang Rai, showed the same patterns of the ratio; they contained high amounts of baicalin (9–11% *w*/*w* of the extracts), some amounts of oroxin B, chrysin-7-*O*-glucuronide, chrysin and baicalein (1–10% *w*/*w* of the extract) and a low amount of oroxin A (<1% *w*/*w* of the extract). It was noted that the seed extract from Lampang province contained a lower amount of most of the compounds but a higher amount of chrysin-7-*O*-glucuronide than the seed extract from Chiang Rai province, which suggests that the collection locations could affect the amounts of phytochemicals. The dry pod coat also contained baicalin as a major compound (around 1% *w*/*w* of the extract) with small amounts of the others, suggesting the same chemical pattern as the mature green fruit. Oroxylin A could be detected only in the pod coat and green mature fruits extracts, of which the amounts were around 1% *w*/*w* of the extracts. Therefore, this compound might be used as a marker for indicating the maturity of the fruits of *O. indicum*. This knowledge could be useful for the quality control of raw materials and herbal products from different plant parts of *O. indicum,* via either the qualitative analysis of the chromatographic profiles or the quantitative analysis of the active compounds.

The results from this study support our previous suggestion that the three unidentified peaks which could be found in the extracts, fractions and crystals of the seeds and the fruits of *O. indicum* could be oroxin A, oroxin B and chrysin-7-*O*-glucuronide [13]. Moreover, two more compounds were isolated and identified; the first one was oroxylin A, which was found in the mature fruit and the pod coat extract, and the other was 5-hydroxymethylfurfural, which was found in the young fruit extract for the first time for both of them. The quantitative analysis of the flavones in this study showed that the results correspond to our previous study reporting the quantitative analysis of baicalin, baicalein and chrysin in some extracts, precipitates and crystals of *O. indicum* [13]. The results corresponded to our current experiment, that extracts from the seeds from Lampang province contained high amounts of baicalin (around 6% *w*/*w* of the dried extract), with some amounts of baicalein and chrysin (around 1% *w*/*w* of the dried extract), while extracts from the young fruits collected from the Nakhon Pathom and Chiang Rai provinces contained low amounts of all three compounds (≤1% *w*/*w* of dried extract) [13].

The flower extract also showed low amounts of all compounds (<1% *w*/*w* of the dried extract) [13]. However, the total phenolic contents between the seed and the young fruit extracts were not much different (4–6 g% GAE in the extracts) [13]. It was reported that the peak areas corresponding to oroxin B, oroxin A and chrysin-7-*O*-glucuronide showed moderate correlations of antioxidant activities and high correlations of inhibitory effects against *Streptococcus aureus*, *Streptococcus suis* and *Pseudomonas aeruginosa* in extracts from *O. indicum* [13].

### 2.3. Quantitative Analysis of Multi-Components Using Single Marker (QAMS) Method for Simultaneous Determination of Flavonoids Contents

The relative conversion factors (RCF) for all the flavonoid standards at 285 nm are listed in Table 4. It can be seen that the response factors at 285 nm for each flavonoid were in the range of 0.21 to 3.01. To select a suitable single marker for the QAMS method, the standard method difference (SMD) between the QAMS method and the external standard method of each compound was calculated as Equation (3). As shown in Table 5, baicalin was selected as the single marker for the QAMS method, since the % SMD of the other compounds were acceptable as not more than 6.8. Under the described HPLC conditions, the conversion factors for the other flavonoids are sequenced as oroxin B > chrysin-7-*O*-glucuronide > oroxylin A > chrysin > baicalein > oroxin A, at 285 nm.

A previous study revealed that baicalin can be used as single marker or reference standard for the HPLC method, utilizing the single reference standard (SRS) analysis of seven flavones including oroxin B, chrysin-7-*O*-gentiobioside, oroxin A, baicalin, baicalein, chrysin-7-*O*-glucuronide and chrysin in the seed samples and commercial products of *O. indicum,* compared with the multiple reference standard (MRS) [39]. No statistical difference was found between the data obtained from the two methods [39]. Oroxin B was also used as the internal reference substance to calculate the amounts of the other flavones, oroxin A, baicalin, chrysin-7-*O*-glucuronide, baicalein and chrysin in the *O. indicum* samples in the QAMS analysis, which showed no significant difference compared to the external standard method [29]. Another previous study reported the use of baicalein as the internal reference standard for the determination of the contents of scutellarin, hispidulin and biochanin A in several parts of *O. indicum* including the leaves, flowers, bark and seed pods from different origins [40]. The results suggested no major difference between the contents from the QAMS and ESM methods [40].

## 3. Materials and Methods

### 3.1. Plant Materials

The seeds of *O. indicum* were purchased from the Lampang and Chaing Rai provinces in 2018 and 2019, respectively (voucher nos. OI-LPG-S01-2018 and OI-CRI-S01-2019, respectively). The young fruits were purchased from Lampang province in 2018 (voucher no. OI-LPG-YF01-2018) while the green mature fruit and the dry pod coat were purchased from Chiang Rai province in 2019 (voucher nos. OI-CRI-MF01-2019 and OI-CRI-PC01-2019, respectively). All voucher plant specimens were kept at the Department of Pharmacognosy, Faculty of Pharmacy, Mahidol University, Bangkok, Thailand. The plant samples were identified according to their botanical and taxonomical characteristics using the identification key presented in *Flora of Thailand* [41]. The plant samples were cleaned and dried in a hot-air oven (50 °C) for 6 h and powdered using an electronic mill (20-mesh sieve).

### 3.2. Preparation of Plant Extracts

Each powdered plant sample was separately macerated with 96% ethanol (plant/solvent ratio of 1:20 *w*/*v*) using an electric flask shaker (Wisd Laboratory Instruments, Wertheim, Germany) for 6 h. The extraction solution was filtered after it was stored for 12 h at room temperature. Each extraction process was repeated three times. The extraction solutions were then combined, filtered and evaporated using a boiling water bath to yield the dried extracts.

### 3.3. Identification and Isolation of Flavones

#### 3.3.1. Identification of Flavones via HPLC-MS

Applied from Sithisarn et al., 2021, the analysis of flavones in the *O. indicum* extracts was performed using Agilent Tech 1260 Infinity II with a 1260 DAD and 1260 vial sampler (Agilent, Santa Clara, CA, USA). A YMC C_18_ column (150 mm × 4.6 mm, 3 µm particle size) from YMC (YMC, Kyoto, Japan) was used for the identification of the flavones. Gradient elution was performed with water/0.1% formic acid (solvent A) and acetonitrile (solvent B) at a constant flow rate of 800 µL/min. The composition was changed from 70% A to 10% A in 20 min. The column temperature was 40 °C, with an injection volume of 5 µL. UV detection was performed at 285 nm. A mass spectrometric analysis of the extracts from *O. indicum* was conducted using a mass spectrometer detector (G6125B) with a gas temperature of 325 °C, drying gas of 12.0 L/min and nebulizer pressure of 1294 Torr. The retention times, UV and mass spectra of the obtained peaks were compared with the reference standards.

#### 3.3.2. Isolation of Flavones in *O. indicum* Extract

From the HPLC analysis, it was found that the pod coat and the mature green fruit extracts contained an unknown peak with a retention time around 27 min; therefore, the isolation of this compound was conducted via liquid–liquid extraction and column chromatography. One-dimensional and two-dimensional nuclear magnetic resonance (NMR) spectroscopic techniques and an LC-MS analysis were performed for structure elucidation.

##### 3.3.2.1. Isolation and Identification of Compound OI1

The LC-MS analysis of the *O. indicum* extracts from various plant parts including the seeds, the young fruits, the mature fruits and the dry pod coat suggested that there are six major flavones in the extract. However, an unknown peak at the late stage of the HPLC chromatograms of the mature fruit and dry pod coat extracts showed up, which could not be found in the other extracts. To identify this peak, the pod coat extract of *O. indicum* (10.3 g) was suspended in distilled water (100 mL) and extracted using continuous liquid–liquid chromatography with petroleum ether (200 mL) for 1 h, then the petroleum ether part was collected, and the process was repeated. Finally, the petroleum ether parts were combined. The remaining aqueous part was then extracted with ethyl acetate and *n*-butanol, respectively, using the same procedure. The obtained petroleum ether, ethyl acetate, *n*-butanol and aqueous fractions were dried using rotary evaporator and were analyzed using thin layer chromatography (TLC), using a silica gel GF_254_ precoated plate and ethyl acetate/toluene/formic acid, 2.5:2.5:0.75% *v*/*v,* as the solvent system. The TLC analysis of all fractions was monitored under UV 254, UV 366 and an NP spray reagent under UV 366 nm. The petroleum ether fraction (100 mg) was dissolved in 3 mL of methanol, filtered, and the solution was submitted to column chromatography (CC) using Sephadex LH20 as the stationary phase and dichloromethane/acetone, 85:12 *v*/*v,* as the mobile phase. Thirty-seven fractions (2 mL) were collected and monitored via TLC using the analytical conditions as mentioned above; fractions with similar TLC patterns were combined. Six combined fractions were obtained. Fraction 5 (16 mg) showed the major quenching spot under UV 254 nm at the Rf value of 0.68. This spot appeared as dark spots under UV 366 and the NP spray reagent under UV 366 nm. The HPLC-DAD analysis of fraction 5, using the HPLC method from Section 3.4, showed a main peak with a retention time of 27.82 min. The online UV spectra exhibited maxima at 270 and 316 nm. This fraction was assigned as compound OI1 (0.16% *w*/*w* of the extract) which appeared as a yellow solid; it was then structurally analyzed using the NMR spectroscopic technique and LC-MS, using the analytical condition as mentioned in Section 3.3.

The ^1^H-NMR data (400.13 MHz, DMSO-*d*_6_) of compound OI1 were assigned on the basis of the chemical shifts, splitting patterns and integration values. The signals at δ 8.07 (1H, m), 7.58 (1H, m), 7.61 (1H, m), 7.58 (1H, m) and 8.07 (1H, m) ppm were assigned to H-2′, H-3′, H-4′, H-5′ and H-6′ of ring B, respectively. The proton signal at δ 6.64 (1H, s) ppm was assigned to H-8 of ring A, while the signal at δ 6.97 (1H, s) was assigned to H-3 of ring C. The signals at δ 12.93 (1H, s) and 11.78 (1H, s) ppm indicated protons of OH groups at C-5 and C-7 of ring A, while a methoxy proton of the methoxy substitute group at C-6 of ring A was found at δ 3.76 (3H, s). The proton signals corresponded to the signals of oroxylin A in the reported literature [42]. The ^13^C NMR (100.61 MHz, DMSO-*d*_6_) of compound OI1 showed 16 signals ascribed to one methoxy carbon (OCH_3_), 59.95 (CH_3_ at C-6); seven methine carbons (CH), 94.41 (C-8), 104.67 (C-3), 126.40 (C-2′), 129.14 (C-3′), 132.01 (C-4′), 129.14 (C-5′) and 126.40 (C-6′); and eight quaternary carbons (C), 163.21 (C-2), 182.27 (C-4), 152.75 (C-5), 131.48 (C-6), 157.69 (C-7), 152.58 (C-9), 104.34 (C-10) and 130.76 (C-1′). Nuclear Overhauser effect spectroscopy (NOESY) showed signals indicating the relationship between the proton of the OH group at C-3 of ring C and the proton at C-6′ of ring B, while correlation spectroscopy (COSY) suggested that the signals indicated a relationship between the proton at C-3′ to the protons at C-2′ and C-4′. The heteronuclear single-quantum correlation spectroscopy (HSQC) and heteronuclear multiple-bond correlation spectroscopy (HMBC) showed that the signals indicated a relationship between protons and carbons, including methoxy protons (OCH_3_) at C-6 to C-6, hydroxy protons (OH) at C-5 to C-6 and C-10, protons at C-8 to C-6, C-7, C-9 and C-4, protons at C-3 to C-4, C-9, C-10 and C-1′, protons at C-2′ to C-2 and protons at C-6′ to C-2. The LC-MS analysis of compound OI1 using the method as mentioned in Section 3.3 revealed a molecular ion peak at 285 [M+H]^+^ and 283 [M-H]^−^, suggesting a molecular weight of 284.

##### 3.3.2.2. Isolation and Identification of Compound OI2

There was a major peak which appeared only in the early part of the HPLC chromatogram of the young fruit extract of *O. indicum*. Therefore, the young fruit extract (10.1 g) was suspended in distilled water (100 mL) and was extracted using continuous liquid–liquid chromatography with petroleum ether (200 mL) for 1 h; then, the petroleum ether part was collected. The process was repeated and the petroleum ether parts were combined. The remaining aqueous part was then extracted with ethyl acetate and *n*-butanol, respectively, using the same procedure, yielding the corresponding fractions. They were dried using a rotary evaporator and subjected to TLC analysis using a silica gel GF_254_ precoated plate and ethyl acetate/toluene/formic acid, 2.5:2.5:0.75% *v*/*v,* as the solvent system. The TLC analysis of all fractions was monitored under UV 254, UV 366 and an NP spray reagent under UV 366 nm. The ethyl acetate fraction (100 mg) was then dissolved in 3 mL of methanol, filtered, and the solution was submitted to CC using Sephadex LH20 as the stationary phase and methanol as the mobile phase. Thirty-seven fractions (3 mL) were collected and monitored via TLC using the analytical conditions as mentioned above. Fractions with similar TLC patterns were combined. Seven combined fractions were obtained. Fraction M2 showed a major quenching spot under UV 254 nm at the Rf value of 0.42. This spot appeared as dark spots under UV 366 and the NP spray reagent under UV 366 nm. Fraction M2 (53 mg) was then dissolved in 1 mL methanol/water 1:1 *v*/*v*, filtered, and the solution was submitted to CC using Sephadex LH20 as the stationary phase and methanol/water 1:1 *v*/*v* as the mobile phase. Two hundred fractions (1 mL) were collected and monitored via TLC using the analytical conditions as mentioned above, and fractions with the similar TLC patterns were combined. Six combined fractions were obtained. Fraction M2.3 (9.6 mg) showed a major quenching spot under UV 254 nm at the Rf value of 0.42. This spot appeared as dark spots under UV 366 and the NP spray reagent under UV 366 nm. Fraction M2.3 was then analyzed via HPLC analysis, as mentioned in Section 3.4, with the adaptation of using 0.1% formic acid in water as eluent A and methanol as eluent B. The gradient elution of the HPLC analysis was performed as 0–10 min, 5% B; 10–12 min, linear gradient from 5 to 13.5% B; 12–20 min, linear gradient from 13.5 to 25% B; 20–30 min, linear gradient from 25 to 90% B; 30–33 min, linear gradient from 90 to 5% B; 33–40 min, 5% B. The column temperature was 40 °C, with an injection volume of 5 µL. UV detection was performed at 285 nm. The HPLC_DAD analysis of fraction M2.3 showed a major peak with a retention time of 14.61 min. The UV spectrum exhibited a maximum of 284 nm. This fraction was assigned as compound OI2 (0.10% *w*/*w* of the extract) which appeared as an off-white solid. It was then structurally analyzed using the NMR spectroscopic technique and LC-MS, using the analytical condition as mentioned in Section 3.3.

The ^1^H-NMR data (600.19 MHz, MeOH-*d_4_*) of compound OI2 were assigned on the basis of the chemical shifts, splitting patterns and integration values. The signals were found at δ: 9.54 (s, 1H, C(O)H-1, aldehyde); 7.38 (d, 1H, CH-3 furan, J = 3.5 Hz); 6.58 (d, 1 H, CH-4 furan, J = 3.5 Hz); 4.61 (s, 2H, CH_2_-6). The ^13^C NMR (150.93 MHz, MeOH-*d_4_*) of compound OI2 showed 6 signals at δ: 179.42 (C-1(O)H, aldehyde); 163.21 and 153.91 (C-5, C-2, furan, respectively); 124.78 and 110.88 (C-3H, C-4H, furan, respectively); 57.63 (C-6H_2_OH). The HSQC and HMBC showed that the signals indicated a relationship between protons and carbons, including the aldehyde proton at C-1 to C-2, the proton at C-3 to C-1, C-2 and C-4, the proton at C-4 to C-2, C-3 and C-5, and the proton at C-6 to C-4 and C-5. The proton and carbon NMR signals corresponded to the signals of 5-hydroxymethylfurfural in the reported literature [43]. The LC-MS analysis of compound OI2 using the method as mentioned in Section 3.3 revealed a molecular ion peak at 127 [M+H]^+^ suggesting a molecular weight of 126.

### 3.4. Quantitative Analysis of Flavone Contents

For the quantitative analysis of the 7 major compounds in the *O. indicum* extracts, the HPLC analysis was optimized and validated using the method applied from Sithisarn et al., 2021 [13], using the Shimadzu LC-20AD XR (Shimadzu, Tokyo, Japan), the YMC Pack Pro C18 (250 × 4 mm, 3 µm particle size) from YMC (YMC, Kyoto, Japan), with the SPD-M20A DAD and an auto-sampler (Shimadzu, Tokyo, Japan). The mobile phases consisted of 0.1% formic acid in water (*v*/*v*) (eluent A) and 10% methanol in acetonitrile (eluent B) at a constant flow rate of 0.6 mL/min. The gradient was as follows: 0–7 min, 30% B; 7–15 min, linear gradient from 30 to 33% B; 15–22 min, linear gradient from 33 to 56% B; 22–23 min, linear gradient from 56 to 90% B; 23–28 min, 90% B; 28–29 min, linear gradient from 90 to 30% B; 29–38min, 30% B. The column temperature was 40 °C, with an injection volume of 5 µL. UV detection was performed at 285 nm.

The linearity for standard oroxin B (Chengdu Biopurify Phytochemicals Ltd., Chengdu, China), baicalin (Chengdu Biopurify Phytochemicals Ltd., Chengdu, China), oroxin A (Chengdu Biopurify Phytochemicals Ltd., Chengdu, China), baicalein (Chengdu Biopurify Phytochemicals Ltd., Chengdu, China), chrysin-7-*O*-glucuronide (Chengdu Biopurify Phytochemicals Ltd., Chengdu, China), chrysin (Chengdu Biopurify Phytochemicals Ltd., Chengdu, China) and oroxylin A (Chengdu Biopurify Phytochemicals Ltd., Chengdu, China) was determined via the analysis of five to six different concentrations, each injected in triplicate. The peak purity was investigated using a DAD for all peaks of interest. The precision of the method for oroxin B, baicalin, oroxin A, baicalein, chrysin-7-*O*-glucuronide and chrysin was evaluated by analyzing six independently prepared solutions of the seed extract from Lampang province (2.31 mg/mL) on three different days. The peak area of each standard compound was determined, and the relative standard deviation percent (%RSD) was calculated. The dry pod coat extract (1.37 mg/mL) was used for evaluation of the precision of oroxylin A using the same procedure. The accuracy of the method was confirmed via the determination of the recovery. The recovery of the seven standard compounds, oroxin B, baicalin, oroxin A, baicalein, chrysin-7-*O*-glucuronide, chrysin and oroxylin A, was performed on the seed extract from Lampang province, spiked with three concentrations of standard mixture. The validated HPLC analytical method was then applied for the quantitative analysis of all seven compounds in the *O. indicum* extracts. The system suitability of the analytical method, including the tailing factor, theoretical plate, the %RSD of the peak area and the SD of the retention time, was also investigated.

### 3.5. Quantitative Analysis of Multi-Components Using Single Marker (QAMS) Method for Simultaneous Determination of Flavonoids Contents

When a single reference is used to determine the concentration of other compounds in a sample, the concentration of each compound (Ci) should be calculated using the ratio between the peak area of the compound in sample solution (Ai) and the peak area of the chosen reference compound in a standard solution in a unit concentration (Ak/Ck), and then calibrated using the relative conversion factor (RCF).

(1) $$Ci=\frac{Ai}{Ak/Ck}\times RCF$$

The relative conversion factor (RCF) for each flavonoid was calculated as the ratio of the peak areas in a unit concentration between the standard substance (Ak/Ck) and the analyte (Ai/Ci):

(2) $$RCF=\frac{Ak/Ck}{Ai/Ci}$$

To calculate the conversion factors, firstly, three series of standard mixture solutions were prepared and analyzed using the developed HPLC method; then, the ratio at each concentration level of the three standard mixture solutions were calculated using Equation (2); finally, the relative conversion factor of each flavonoid was obtained as the mean values calculated from the triplet of the five concentrations.

For the comparison of a new QAMS method with the external standard method, the standard method difference (SMD) was computed from the results of the analysis of the young fruit extract from Lampang province, and the green mature fruit and seed extracts from Chiang Rai province, according to the following equation:

(3) $$SMD=\frac{C_{ES}-C_{QAMS}}{C_{ES}}\times 100$$

where C_ES_ and C_QAMS_ represent the concentrations of the flavonoids assayed in the *O. indicum* extracts using the external standard method and the QAMS method, respectively.

### 3.6. Statistical Analysis

All data are reported as means ± the standard deviation of the triplicates. An analysis of variance (ANOVA) was used to compare the means of the contents of most compounds, except for the content of oroxylin A, for which an independent sample *t*-test was used at the least significant difference of *p* < 0.05. All analyses were performed using Microsoft Excel (Microsoft Office Professional Plus 2016—Microsoft Corporation, Redmond, DC, USA).

## 4. Conclusions

Three flavones, oroxin A, oroxin B and chrysin-7-*O*-glucuronide, were identified from *O. indicum* extracts. Oroxylin A was isolated and identified from the pod coat extract, while 5-hydroxymethylfurfural was isolated and identified from the young fruit extract. The HPLC analysis was optimized and validated for the simultaneous quantitative analysis of seven flavones in the young fruit, the green mature fruit, the pod coat and the seed extracts of *O. indicum* for the first time. Baicalin was found to be the major compound in all extracts. The flavone contents were low in the young stages of the fruits, especially in the young fruits that are common edible part. The green mature fruits and pod coats, two parts that are not edible, showed similar phytochemical ratios. The seeds are a rich source of flavones, since they contained up to 11% *w*/*w* of baicalin in the dry extracts, while oroxin A showed the lowest amount of all the investigated samples. Oroxylin A could be used as a marker to indicate the maturity of *O. indicum* fruits, such as with the green fruits in the mature stage or the dry pod coats, while 5-hydroxymethylfurfural could be used as a marker for the young fruits. The QAMS method was firstly developed, and baicalin was found to be suitable for use as single marker to calculate the contents of all flavones in the *O. indicum* extracts. The data of the flavone amounts obtained from the HPLC and QAMS methods were compared for the first time, which suggested no difference between these two methods.

## Figures and Tables

**Table 1:** Retention times (RT), UV maxima from LC-online-UV spectra (λ_max_) and molecular mass ions [M+H]^+^/[M-H]^−^ of 7 major compounds in *O. indicum* samples.

Peak No.	Compound	RT (min.)	λ_max_ (nm)	*m*/*z* [M+H]^+^/[M-H]^−^
1	Oroxin B (**1**)	6.73	278, 316	595.2/593.0
2	Oroxin A (**3**)	8.81	278, 316	433.2/431.0
3	Baicalin (**2**)	10.91	276, 316	447.2/445.0
4	Chrysin-7-*O*-glucuronide (**4**)	14.67	268, 306	431.2/428.8
5	Baicalein (**5**)	17.78	272, 292sh, 318	271.2/268.8
6	Chrysin (**6**)	21.67	268, 314	255.0/252.8
7	Oroxylin A (**7**)	22.16	270, 316	285.2/283.0

**Table 2:** Calibration curves, accuracy and precision of HPLC method for determination of flavonoids in *O. indicum* extract.

Compound	Range (µg/mL)	r	Added Concentration (µg/mL)	% Recovery	% RSD of Precision	
					Repeatability	Intermediate Precision
OB (**1**)	0.65–500	0.9999	5.2–20	95–103	0.1–0.8	0.6
BL (**2**)	1–132	0.9978	13.2–52.8	96–101	0.1–0.2	0.3
OA (**3**)	0.7–500	0.9987	5.6–22.4	101–105	0.1–0.4	1.5
C7 (**4**)	0.7–56	0.9992	5.6–22.4	97–104	0.1–0.8	1.3
BE (**5**)	1–92	0.9994	9.2–36.8	97–101	0.2–0.9	0.8
C (**6**)	2–160	0.9979	16.0–24.0	101–104	0.2–0.3	0.7
OX (**7**)	2–160	0.9999	14.8–59.2	101–104	0.4–0.5	0.6

**Table 3:** Quantitative analysis of 7 compounds in *O. indicum* extracts via HPLC.

Extract	Content (% *w*/*w* in the Extract) *						
	OB (1)	BL (2)	OA (3)	C7 (4)	BE (5)	C (6)	OXA (7)
YFL	n.d.	0.41 ± 0.00 ^a^	0.08 ± 0.00 ^b^	n.d.	0.53 ± 0.00 ^c^	0.96 ± 0.00 ^b^	n.d.
GFC	0.54 ± 0.00 ^a^	2.77 ± 0.00 ^c^	0.72 ± 0.00 ^e^	0.69 ± 0.00 ^b^	0.79 ± 0.00 ^d^	1.11 ± 0.00 ^d^	1.35 ± 0.00 ^b^
DPC	n.d.	1.44 ± 0.00 ^b^	0.06 ± 0.00 ^a^	0.04 ± 0.00 ^a^	0.09 ± 0.00 ^a^	0.66 ± 0.00 ^a^	1.20 ± 0.00 ^a^
SC	10.90 ± 0.07 ^c^	11.47 ± 0.03 ^e^	0.51 ± 0.01 ^d^	1.08 ± 0.01 ^c^	2.42 ± 0.02 ^e^	2.57 ± 0.02 ^e^	n.d.
SL	2.90 ± 0.01 ^b^	9.12 ± 0.01 ^d^	0.14 ± 0.00 ^c^	2.47 ± 0.00 ^d^	0.27 ± 0.00 ^b^	1.03 ± 0.00 ^c^	n.d.

YFL = young fruit extract (Lampang), GFC = green fruit extract (Chiang Rai), DPC = dry pod coat extract (Chiang Rai), SC = seed extract (Chiang Rai). SL = seed extract (Lampang); OB = oroxin B (**1**), BL = baicalin (**2**), OA = oroxin A (**3**), C7 = chrysin-7-*O*-glucuronide (**4**), BE = baicalein (**5**), C = chrysin (**6**), OXA = oroxylin A (**7**); BL = baicalin, OA = oroxin A, C7 = chrysin-7-*O*-glucuronide, C = chrysin, OXA = oroxylin A; n.d. = cannot be detected. * Different letters in the same column indicate significant differences (*p* < 0.05).

**Table 4:** Relative conversion factors (RCF) and standard deviation (SD) values of flavonoids of *O. indicum* extracts.

Other Compound	Reference Compound													
	OB (1)		BL (2)		OA (3)		C7 (4)		BE (5)		C (6)		OXA (7)	
	RCF	SD	RCF	SD	RCF	SD	RCF	SD	RCF	SD	RCF	SD	RCF	SD
OB (**1**)	-		0.65	0.05	2.98	0.19	1.08	0.08	1.97	0.22	1.78	0.13	1.69	0.12
BL (**2**)	1.54	0.11	-		4.56	0.31	1.65	0.13	3.01	0.35	2.73	0.21	2.6	0.2
OA (**3**)	0.33	0.02	0.21	0.02	-		0.35	0.03	0.65	0.07	0.59	0.04	0.55	0.04
C7 (**4**)	0.93	0.07	0.61	0.05	2.78	0.2	-		1.83	0.22	1.66	0.13	1.58	0.12
BE (**5**)	0.52	0.06	0.34	0.04	1.53	0.18	0.55	0.07	-		0.92	0.11	0.87	0.11
C (**6**)	0.56	0.04	0.37	0.03	1.67	0.11	0.61	0.05	1.1	0.13	-		0.96	0.07
OXA (**7**)	0.59	0.04	0.39	0.03	1.77	0.11	0.64	0.05	1.16	0.13	1.06	0.08	-	

**Table 5:** Comparison for the contents (% *w*/*w*) of markers measured via EMS and QAMS in young fruit, green fruit and seed extracts of *O. indicum*.

Compound	Young Fruit			Green Fruit			Seed		
	Content, %		SMD, %	Content, %		SMD, %	Content, %		SMD, %
	EMS	QAMS		EMS	QAMS		EMS	QAMS	
Oroxin B (**1**)	n.d.	n.d.	-	0.54	0.52	3.7	7.63	7.43	2.6
Baicalin (**2**)	0.41	- ^a^	-	2.77	- ^a^	-	9.01	- ^a^	-
Oroxin A (**3**)	0.08	0.08	3.80	0.72	0.72	0.2	0.34	0.36	4.5
Chrysin-7-*O*-glucuronide (**4**)	n.d.	n.d.	-	0.68	0.67	1.7	0.74	0.73	2.5
Baicalein (**5**)	0.53	0.57	6.8	0.79	0.85	6.7	1.82	1.93	6.8
Chrysin (**6**)	0.92	0.96	5	1.11	1.18	6.1	1.91	1.90	0.4
Oroxylin A (**7**)	n.d.	n.d.	-	1.35	1.34	1.3	n.d.	n.d.	-

^a^ Baicalin was used as the single marker in the QAMS method.

## Data Availability

Data sharing not applicable.
